# Supplementary material for: Transcranial Direct Current Stimulation Enhances Motor Performance by Modulating Beta-Phase Synchronization in the Sensorimotor Network: A Preliminary Study
Source: Brain Sci. 2025 Mar 7;15(3):286. doi: 10.3390/brainsci15030286 (PMC11940359; doi:10.3390/brainsci15030286)

Supplementary

S1. Full-band spectrogram of neural activity recorded across conditions (averaged across participants).

Although the sham condition visually exhibits higher alpha amplitude, statistical analyses confirmed no significant differences in alpha power.

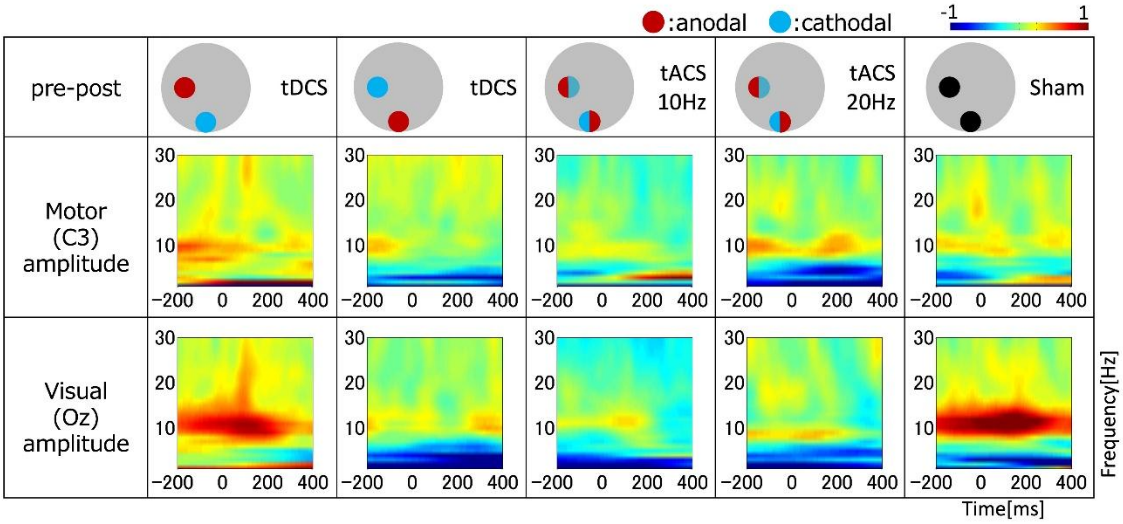

Supplement: Supplementary file 1 [file brainsci-15-00286-s001.zip › brainsci-3472959-supplementary.pdf]
